# Regulation of Microstructure and Properties of Konjac Glucomannan Gels via Ethanol Under Low-Alkali Conditions

**DOI:** 10.3390/gels12010083

**Published:** 2026-01-17

**Authors:** Meiqiu Xu, Hongtao Du, Solairaj Dhanasekaran, Yin Jia, Yange Ren, Hong Chen, Wei Xu

**Affiliations:** 1 College of Food and Drug, Luoyang Normal University, Luoyang 471934, China; xmq1963191@lynu.edu.cn (M.X.); r18839536990@163.com (Y.R.); 2 College of Life Sciences, Yan’an University, Yan’an 716000, China; 3 Department of Biotechnology, PSGR Krishnammal College for Women, Coimbatore 641004, India; solaibt@hotmail.com; 4 College of Life Science, Xinyang Normal University, Xinyang 464000, China; jy928357397@163.com

**Keywords:** konjac glucomannan, ethanol, gel, low alkali

## Abstract

Despite their potential, alkali-treated konjac glucomannan (KGM) gels are limited by excessive brittleness and a lack of eco-friendly synthesis methods, creating an urgent need for more durable and ‘green’ alternatives. In this study, highly stable KGM gels were constructed under low-alkali conditions by adjusting the ethanol content. The results showed that intermolecular hydrogen bonding and hydrophobic interactions were enhanced with increasing ethanol concentration (0–20% *v*/*v*) under low-alkaline conditions. The physicochemical properties of KGM gels showed dynamic improvement, with denser micro-network morphology and simultaneous enhancement of thermal stability. However, the addition of a high ethanol concentration (20% *v*/*v*) tended to trigger local aggregation, disrupting the gel network structure. At an ethanol addition of 15%, the hydrogen bonding and hydrophobic interactions of KGM gels reached an optimal equilibrium, exhibiting the most compact gel network and excellent resistance to deformation. This study reveals the regulation of the microstructure and macroscopic properties of KGM gels by ethanol, which provides theoretical support for the construction of high-performance KGM gels under low-alkali conditions.

## 1. Introduction

Gels are a special type of semi-solid dispersion system found in nature, internally connected by molecules that exhibit a three-dimensional (3D) network structure [1,2]. Over the past decade, polysaccharide-based hydrogels have been widely studied because of their biocompatibility, biodegradability, and ability to act as drug delivery systems. Among these, konjac glucomannan (KGM) has become a research hotspot in the field of polysaccharide gels due to its excellent gelling properties and the health benefits of dietary fibers. KGM gels are already known for their versatile applications in the food industry, such as texture modifiers, fat analogues, thickeners, carriers, edible films and coatings [3,4].

KGM is a non-ionic, water-soluble polysaccharide formed by linking D-glucose and D-mannose with β-1,4 and β-1,3 glycosidic bonds, with acetyl groups randomly distributed along the main chain [5,6]. Depending on the preparation method, KGM can form gels in two different ways, namely, it can form a gel that melts when heated (alkali-induced) or one that remain solid even at high temperatures. These stable, heat-resistant gels are created through methods, such as heating, enzyme use enzymes, or molecular reorganization in a neutral environment. These non-alkali KGM gels have been extensively investigated and exhibit promising structural stability for biomedical and functional material applications [7]. The superior gelling properties of KGM are dependent on alkali and thermal treatments, which help remove acetyl groups from the chain. This deacetylation allows D-glucose and D-mannose chains to aggregate into a strong thermoreversible complex [8]. However, its gelation process usually relies on strong alkaline conditions (pH 11–13), and the process has many limitations in practical use. On the one hand, strong alkaline treatment is prone to cause quality defects, such as residual alkaline flavor in the product, which affects its organoleptic acceptability in food products, and high-alkaline gels are susceptible to huge water loss over time. However, the discharge of alkaline waste liquid during gel preparation poses a potential environmental pollution risk [9,10]. Although heat-resistant, non-alkali gels solve some problems, they are often difficult to prepare because they require a lot of energy or very specific conditions. This limits their use in semi-solid foods [7]. Consequently, researchers have focused on finding a “middle ground” by creating KGM gels that use minimal alkali to achieve optimal results without the drawbacks of harsh chemicals. Therefore, to solve the problem caused by strong-alkali preparation, it is of great practical value to develop low-alkali KGM gels for better application in the food industry. Moreover, low-alkali KGM gels could be a more attractive substance for healthy foods because KGM is a dietary fiber that promotes gut health, such as fish paste, synthetic soft food, food for the elderly.

Essentially, the aggregation and entanglement of KGM molecular chains are influenced by hydrogen bonds and hydrophobic interactions [11]. Compared to the directional and reversible nature of hydrogen bonds, hydrophobic interactions play a central role in facilitating the tight alignment of the KGM chains. Moreover, the tight intermolecular interactions between KGM required deacetylation, which was accelerated in an alkaline environment. The enhancement of such interactions can be achieved by adjusting the temperature or by introducing specific hydrophilic and hydrophobic excipients, such as sugars, ethanol, or polyols. Notably, the presence of ethanol interacts with the hydroxyl groups of the KGM chains and enhances the intermolecular association under significantly lower alkaline conditions [12]. Previous studies have shown that immersing κ-carrageenan in ethanol solutions of varying concentrations induces significant changes in gel volume and structure, with gel strength increasing as the ethanol concentration increases [13]. In a water–ethanol solution of xyloglucan, alcohol molecules primarily occupy the voids of the water hydration shell in a hydrophobic manner. This causes the molecular chain movement space to decrease, leading to the aggregation and entanglement of polysaccharides, contributing to the formation of xyloglucan gels [14]. Additionally, previously developed ethanol-induced KGM gels form stronger, more compact, more brittle, and thermally stable networks with lower swelling and tunable ion release compared with conventional aqueous/alkali KGM gels [12,15]. However, ethanol clearly alters aggregation and network formation, but the role of ethanol in KGM chain aggregation and structural reorganization is not yet understood and has not yet been optimized for real products, which has motivated further process refinement [11,12,15]. Based on these considerations, it was hypothesized that the addition of moderate ethanol could enhance the hydrophobic interactions between KGM chains and enable the stable formation of gels in low-alkali environment. The addition of ethanol could be a promising approach for developing low-alkali KGM gels. In this study, sodium carbonate was selected as the alkaline agent instead of the more commonly used NaOH because its milder alkalinity and buffering ability allows for the controlled and gradual deacetylation of KGM chains.

Currently, researchers are increasingly interested in developing innovative semi-solid alcohol-containing products and studies on ethanol-induced KGM gel formation [12,15]. For instance, alcohol-based gels were found to protect heat-sensitive or alcohol-soluble active compounds, such as curcumin, and release them in a controlled way to the target [16]. Similarly, hydro-alcoholic gels are being explored for cosmetics and injectable drug delivery applications [17]. However, most of these studies used high doses of ethanol for immersion (20–100% *v*/*v*), and there is a lack of systematic studies on the effect of ethanol concentration on KGM gels. Therefore, this study aimed to develop low-alkali KGM gels using a small-dose ethanol-assisted strategy, which is more practical and sustainable than conventional methods. The effects of different ethanol concentrations on the rheological properties, gel performance and microscopic morphology of low-alkali KGM gels were investigated to provide new ideas for the construction of high-performance KGM gels under low-alkali conditions.

## 2. Results and Discussion

### 2.1. Visual Aspects and Swelling Ratio of KGM Gels

**Figure 1:**
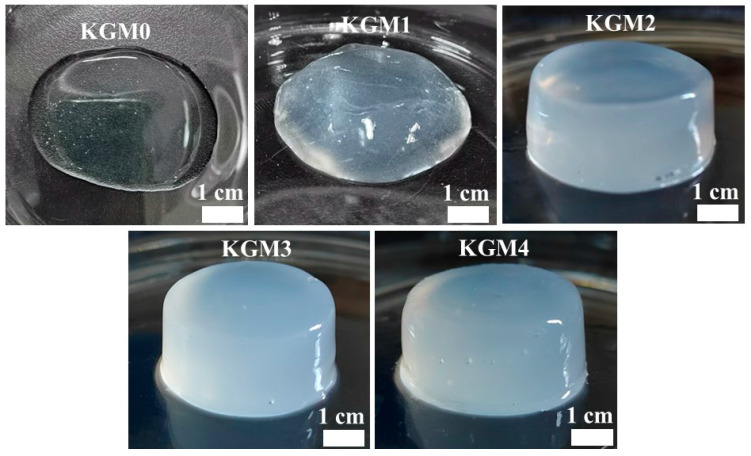
Photographs of KGM gels with different ethanol concentrations (0–20% *v*/*v*) after standing at 25 °C for 3 h. KGM0, KGM1, KGM2, KGM3, and KGM4 correspond to gels prepared with final ethanol concentrations of 0, 5, 10, 15, and 20% (*v*/*v*), respectively.

Figure 1 presents the visual appearance of KGM gels with different ethanol concentrations after 3 h at room temperature. In the absence of ethanol, stable gel formation was absent in KGM0, and high water loss was observed, indicating that a low-alkali environment was insufficient to develop gels. With increasing ethanol concentration, KGM gradually formed polysaccharide gels with uniform texture and better self-supporting properties, suggesting that ethanol plays an important role in enhancing polysaccharide aggregation under low-alkali conditions. Interestingly, increasing the ethanol concentration increased the visual opacity of the KGM gels. This is mainly attributed to the fact that an increase in ethanol concentration promotes water repulsion between polymers and reduces the distance between aggregates [18], resulting in a gradual increase in gel opacity. Ethanol-assisted KGM gels are comparable to alkali-induced KGM gels in terms of shape retention and stability while minimizing alkali usage [8]. These visual appearances provide preliminary evidence of ethanol-induced aggregate formation, which is further investigated using rheological measurements and structural analysis in the following sections.

**Figure 2:**
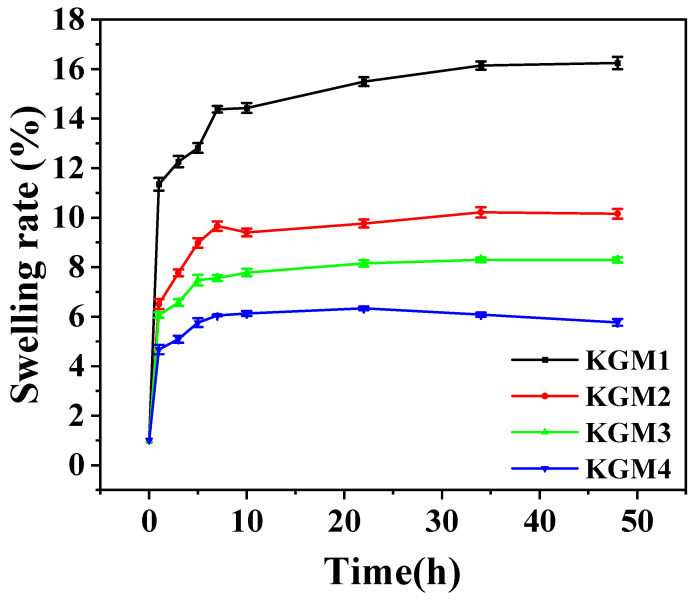
The effect of ethanol concentrations (5–20% *v*/*v*) on swelling kinetics of KGM gels over time. Values are expressed as mean ± SD (*n* = 3). KGM1, KGM2, KGM3, and KGM4 correspond to gels prepared with final ethanol concentrations of 5, 10, 15, and 20% (*v*/*v*), respectively.

The swelling ratio reflects the size and crosslinking density of the gel networks. As shown in Figure 2, all the gels initially exhibited rapid water absorption before ultimately reaching a mass-stable state. Notably, the swelling ratio was negatively correlated with alcohol concentration. As ethanol concentration increased, a densely packed network structure gradually formed within the gel. This narrows the internal channels through which water molecules diffuse into the KGM gel, thereby increasing the internal resistance and consequently reducing the swelling rate [19]. Moreover, a higher ethanol concentration lowers the number of free water molecules around the KGM chains, creating a poor solvent system for the KGM chains. In this highly hydrophobic medium, polymer–polymer interactions are more likely to occur than polymer-water interactions, thereby promoting high chain aggregation and strengthening [20]. Cassanelli et al. further indicated that alcohols serve as fixatives to promote the hardening of network structures [21] and simultaneously generate incoming water pressure during the shrinkage process, thereby reducing the rate of swelling. At 20% ethanol, partial disruption of the gel network occurred, and the reduced swelling rate was likely due to ethanol weakening the competition between water molecules and KGM hydrophilic groups, thus hindering the penetration of water. Moreover, the swelling rate of KGM gels decreased with increasing ethanol fraction, which can be attributed to the poorer solvent quality of ethanol for KGM, resulting in reduced water uptake [15].

### 2.2. Water Holding Capacity (WHC) of KGM Gels

**Figure 3:**
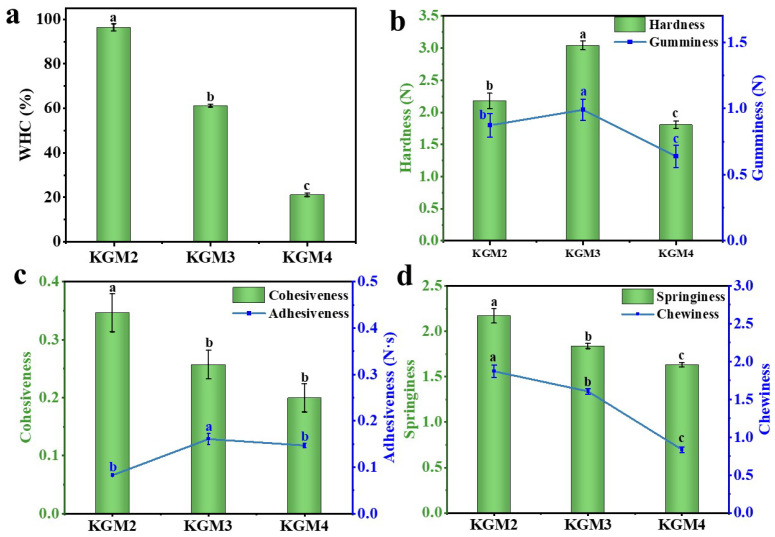
The effect of the ethanol concentrations (10–20% *v*/*v*) on water-holding capacity (WHC) of KGM gels after centrifuged at high speed centrifugation at 8000 rpm, 20 min at 25 °C (**a**) and texture profile analysis parameters (**b**–**d**) of low-alkali KGM gels. Different letters indicate statistically significant differences among samples (*p* < 0.05). KGM2, KGM3, and KGM4 correspond to gels prepared with final ethanol concentrations of 10, 15, and 20% (*v*/*v*), respectively.

Figure 3a shows the changes in the WHC of the gel with different ethanol concentrations (10–20% *v*/*v*). As the ethanol concentration increased, the WHC decreased. The decrease in WHC is attributed to the addition of ethanol reducing the gels polarity. Ethanol weakens the interaction between KGM and water, decreasing the distance between KGM molecules, and making them more likely to approach each other and form hydrogen bonds [22,23]. At the same time, nonpolar regions are exposed, promoting aggregation between hydrophobic segments and enhancing interactions. Consequently, the molecular chains become more tightly entangled, leading to the formation of a highly cross-linked dense network within the gel. Furthermore, ethanol reduces the dielectric constant, which retains the ions inside the gel and enhances polymer–polymer interactions, further tightening the network and limiting water holding [24]. However, this overly dense structure limits the water retention space, causing free water to release and resulting in a reduced WHC [25]. However, at higher ethanol concentrations (20% *v*/*v*), ethanol and water molecules compete in their interactions with the hydrophilic groups of the gel. Ethanol molecules bind to the hydrophilic groups of the gel through their hydrophilic groups, inhibiting the binding of water molecules and leading to a decrease in WHC [9,11,22,26]. At the same time, the gel network became loose and could not effectively lock the water. In contrast, alkali-induced gels exhibited higher swelling ratios and WHC due to their more open network structure, which facilitated a water-rich network [27]. These results indicate that when developing low-alkali KGM gels, the concentration of ethanol should be optimized to achieve a balanced network strength and WHC.

### 2.3. Texture of KGM Gels

To further evaluate the effect of ethanol on the mechanical and textural properties of KGM gels, textural analysis was conducted on KGM gels prepared with varying ethanol concentrations (10–20% *v*/*v*). As shown in Figure 3b–d, moderate ethanol dehydration enhanced the hydrophobic interactions and hydrogen bonding between KGM molecular chains, promoting gel network densification [28]. Hardness, gumminess, and adhesiveness are important textural properties because they positively correlate the gel’s structure to how food feels during biting, chewing, and swallowing, thereby influencing the gel’s swallowing safety, functionality, and consumer acceptance [29]. The hardness, gumminess and adhesiveness of the gel gradually increased and reached a maximum at 15% *v*/*v* of ethanol. However, when the ethanol concentration was further increased to 20% *v*/*v*, the polar environment of the system markedly diminished. Excessively strong hydrophobic interactions led to excessive aggregation and entanglement of the molecular chains, causing a localized collapse of the gel network structure [12,13]. The hardness, cohesiveness, and adhesiveness rapidly declined, consistent with the WHC results. High ethanol concentrations increased the rigidity of the molecular chains and restricted the elastic recovery of the gel network, resulting in a marked increase in brittleness and a reduction in chewability. These changes ultimately impair the textural properties of the gel. Similar to our results, increasing ethanol concentration positively correlates with increased gel hardness and strong shrinkage in alginate, starch, whey, and egg white protein [30,31]. These findings indicate that although ethanol effectively alters the texture of KGM gels under low-alkali conditions, its concentration should be monitored to prevent excessive dehydration, which could negatively affect the mechanical and textural qualities of the gels.

### 2.4. Rheological Properties

**Figure 4:**
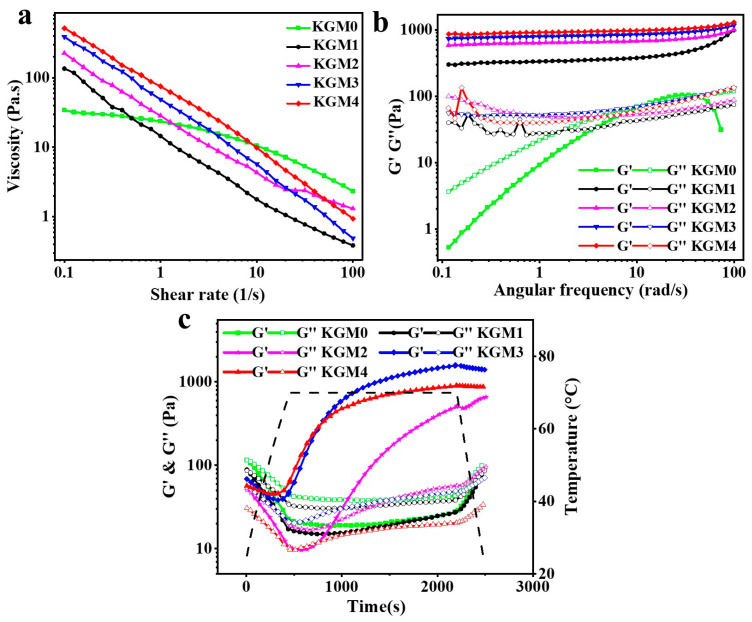
Rheological properties of KGM gels as a function of ethanol concentration (0–20% *v*/*v*). (**a**) Steady-state shear flow curves (apparent viscosity vs. shear rate); (**b**) frequency sweeps; and (**c**) dynamic temperature sweeps showing the thermal stability of the gel networks. The dotted line (**c**) indicted the temperature as a function of time. KGM0, KGM1, KGM2, KGM3, and KGM4 correspond to gels prepared with final ethanol concentrations of 0, 5, 10, 15, and 20% (*v*/*v*), respectively.

Rheological analysis of gels is crucial for characterizing the gel network structure, predicting how a gel flows and deforms under stress, and its stability [32]. The steady-state shear curves (Figure 4a) show that the KGM gel system exhibits typical shear-thinning characteristics at different ethanol concentrations (0–20% *v*/*v*). Viscosity decreased significantly with increasing shear rate. The increasing shear thinning property indicates that the KGM gels are crosslinked with reversible joints, which dissociate under stress and recover upon stress removal [25]. As the shear rate increased, the viscosity decreased markedly. This phenomenon is primarily attributed to the dissociation of entangled gel molecular chains under shear stress, which reduces the flow resistance [33]. The apparent viscosity of the KGM gels increased with increasing ethanol concentration. Increasing ethanol concentration gradually weakened the hydration of the KGM molecules, enhancing the intermolecular interactions and chain entanglement. Restricted chain mobility increases the internal friction and flow resistance within the gel, thereby elevating its viscosity. This shear-dependent viscosity is beneficial for processing, as it allows the gels to flow under shear while maintaining their structural features under normal conditions [34]. Moreover, higher degrees of acetylation in KGM mainly slow the kinetics of gel formation rather than strongly altering the ultimate viscosity of the gel [35].

The frequency scan can indicate the structural integrity and mechanical strength of a sample. The results in Figure 4b show that the KGM0 loss modulus (G″) is greater than the storage modulus (G′), that is at this point, the gel is not formed, consistent with the visual appearance. This result confirmed that a low-alkali environment is insufficient to promote a stable gel network with strong intermolecular interactions. With the addition of ethanol, the gel systems exhibited G′ > G″, indicating a typical solid-like gel behavior, which confirmed the formation of a physically interlinked gel network with strong integrity. With increasing in ethanol concentration, G′ gradually increased. This is because the addition of ethanol weakened the polarity of the system. Ethanol molecules preferentially interacted with water, displacing a portion of the water originally bound to the KGM molecules. This weakened the KGM-water interactions, promoted hydrogen bond rearrangement among KGM molecules, and strengthened the hydrophobic interactions between polymer chains [19,23]. Consequently, the crosslinking density and structural stability of the gel network increased, enhancing its resistance to deformation under external shear stress. These results indicate that ethanol may act as a potential regulator of gel elasticity by modifying the intermolecular interactions and connectivity within the KGM matrix. Although the KGM4 gel retained strong transient mechanical properties under high-frequency shear, its structural uniformity and elasticity were inferior to those of KGM3, indicating a partial disruption of the balance between network rigidity and flexibility. These results indicate that at high ethanol concentrations, KGM chains aggregate quickly in an uneven manner, which creates weak joints that compromise the elasticity of the gel despite high stiffness [36].

Dynamic temperature scanning (Figure 4c) was used to track the formation of KGM gels during ethanol treatment. This analysis provides an understanding of the thermal stability and reversibility of gels, which is important for understanding their behavior during thermal processing [37]. The gelation process consisted of heating, holding, and cooling stages. At the initial stage of warming, thermal energy enhances the mobility of KGM molecular chains, weakening intermolecular physical crosslinks and hydrogen bonding [38]. Partial dissociation of the gel network occurred, as reflected by the simultaneous decrease in G′ and G″. Upon entering the isothermal phase, both G′ and G″ increased significantly, indicating that the gel network began to reform and gradually stabilize. Maintaining a constant temperature during this process provides relatively mild conditions for the reconstruction of the gel network structure. This isothermal phase is important because the reconstruction of the gel network structure is a time-dependent process that allows the chain to form more stable structures. At this stage, the presence of ethanol modulates the balance of intermolecular forces, promoting the rearrangement of intermolecular hydrogen bonds and aggregation of hydrophobic regions. As these interactions intensify, the molecular chains rearrange, continuously strengthening the network structure. Consequently, the overall strength of the gel increased, as indicated by the sustained increase in G′. These observations indicate that ethanol influences gel formation thermodynamically and regulates network reconstruction during thermal treatment. Nevertheless, when the ethanol concentration increased to 20% *v*/*v*, excessively strong intermolecular interactions led to over-crosslinking, resulting in network destabilization and a subsequent decrease in G′. Subsequently, during the cooling phase, the molecular thermal motion is weakened, and hydrogen bonding and hydrophobic interactions are further enhanced [39]. The KGM molecular chains gradually solidified, reinforcing the network structure formed during the constant-temperature phase. Additionally, there is a positive correlation between thermal stability and gel strength, which was evidenced by freezing-thawing experiments conducted in KGM gels [40]. The temperature responsiveness of the KGM gels suggests that the low-alkali-ethanol induced KGM gels are suitable for applications involving repeated heating and cooling. Overall, rheological properties of KGM gels confirmed that ethanol equilibration enhanced the rheological performance, as evidenced by increased storage and loss moduli and a transition toward more elastic, solid-like networks. KGM gels subjected to mild alkali-induced partial deacetylation exhibited particularly high stiffness and stronger physically associated zones, resulting from ethanol-induced intermolecular aggregation dominated by hydrophobic and hydrogen-bonding interactions.

### 2.5. Fourier Transform Infrared Spectroscopy (FTIR) Analysis

**Figure 5:**
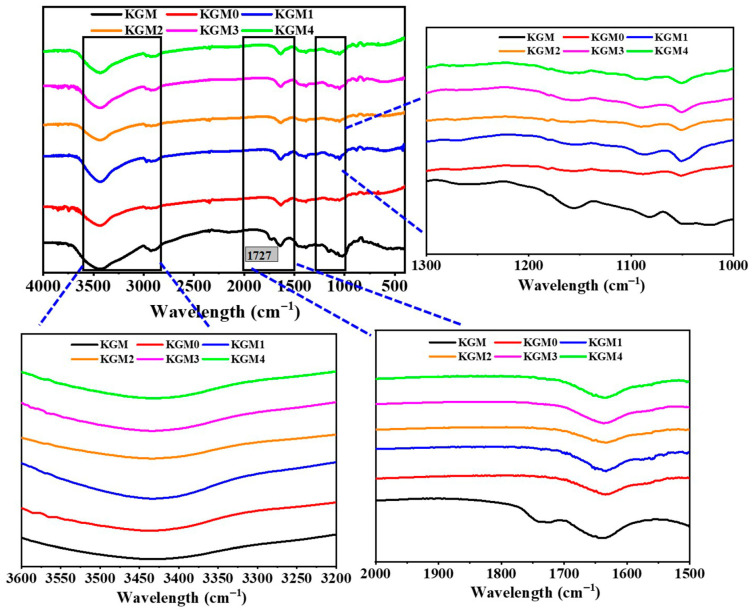
FTIR spectra of KGM gels with different ethanol concentrations. KGM0, KGM1, KGM2, KGM3, and KGM4 correspond to gels prepared with final ethanol concentrations of 0, 5, 10, 15, and 20% (*v*/*v*), respectively. KGM corresponds to polymer powder alone.

FTIR spectroscopy was used to further analyze the chemical bonding changes and molecular structure of the KGM gels under the influence of ethanol. This analysis gives evidence for the molecular-level chain interactions involved in KGM gel formation by ethanol. As shown in Figure 5, for natural KGM, the appearance of broad peaks at 3446 cm^−1^ and 2923 cm^−1^ can be assigned to the stretching vibrations of O-H groups and C-H bonds. The O-H stretching band corresponds to excessive hydrogen bonding and strong hydration of the KGM chains. Characteristic peaks at 1727 cm^−1^ and 1641 cm^−1^ were identified sequentially, which corresponded to the C=O stretching vibration of the acetyl group and intramolecular or intermolecular hydrogen bonding vibration, respectively. These functional groups play an important role in regulating chain plasticity and intermolecular interactions during gel formation [41,42]. Compared with KGM, the spectra of KGM (0–4) showed similar profiles, indicating that ethanol addition did not cause significant changes in molecular structure, which confirms that ethanol influences the physical interactions of KGM backbone rather than causing chemical changes. However, the absorption peak at 1727 cm^−1^ was markedly weakened or even disappeared in KGM (0–4), suggesting that mild alkali-induced partial deacetylation and thereby enhanced intermolecular interactions among KGM molecules [43]. The acetyl groups are supposed to form acetate ions, which dissolve in the aqueous phase and could be removed during gel preparation and sample processing. And further, it can be found that the characteristic peaks at 3446 cm^−1^ and 1641 cm^−1^ of KGM (1–4) were red-shifted with increasing ethanol concentration. This indicates that ethanol weakened the hydration effect of the system, which resulted in enhanced inter- and intramolecular hydrogen bonding [44], restricted molecular chain movement and a denser network structure. This red shift indicates strong and reorganized H-bonds, corresponding to the formation of a compact and interconnected gel network. In addition, the enhancement of the C-H bond stretching vibration at 2980 cm^−1^ reflects that the ethanol addition promotes the aggregation between non-polar groups in the KGM molecules and significantly enhances the intermolecular hydrophobic interactions [19]. With the enrichment of the hydrophobic regions, the entanglement between molecular chains was intensified, thus conferring higher densification and stability to the gel structure. These FTIR results strongly indicate the formation of KGM gels, in which ethanol facilitates dehydration, forces H-bond rearrangement and hydrophobic aggregation, leading to high mechanical strength and structural stability of low-alkali KGM gels.

### 2.6. X-Ray Diffraction (XRD) Analysis

**Figure 6:**
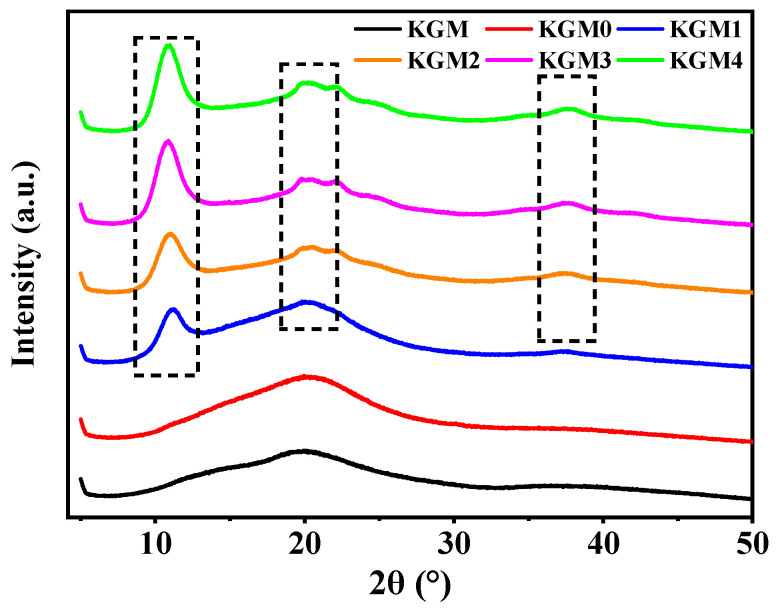
The XRD patterns of KGM gels with different ethanol concentrations (0–20% *v*/*v*). KGM0, KGM1, KGM2, KGM3, and KGM4 correspond to gels prepared with final ethanol concentrations of 0, 5, 10, 15, and 20% (*v*/*v*), respectively. KGM corresponds to polymer powder alone.

To further understand the effect of ethanol on the aggregation structure and crystalline behavior of KGM gels, XRD patterns of KGM gels treated with different ethanol concentrations were obtained (Figure 6). XRD analysis provides knowledge on molecular ordering by uncovering changes at the supramolecular level. KGM exhibited a broad diffuse scattering peak at 2θ = 20°, indicating a typical amorphous structure, which is consistent with the results of Zhang et al. [45]. This amorphous nature of the peak indicates the irregular arrangement of KGM chains in the hydrated state and explains the weak mechanical strength of ethanol-free KGM. With the addition of ethanol, the KGM gel showed new diffraction peaks at 2θ = 10.9° and 37.5°, which were enhanced with the increasing of ethanol concentration, and the peak shapes tended to be sharp (KGM (1–4)). This is related to the addition of ethanol, which induced an ordered rearrangement of the KGM molecular chains. Regularly arranged crystal structures were formed in some regions, and the system gradually changed from the original disordered to ordered state [46]. This molecular arrangement and partial crystallization act as physical crosslinking points in gel network, contributing to increased stability and elasticity. Meanwhile, the broad peak at 2θ = 20° gradually weakened and split into several smaller peaks as ethanol concentration increased, indicating the formation of crystalline regions with different orientations. This peak splitting indicates the presence of a heterogenic structure with increasing ethanol concentrations. The addition of ethanol strengthened hydrogen bonding and hydrophobic interactions between molecular chains, promoting KGM chain aggregation and ordered alignment. As a result, local crystalline structures were formed, leading to the observed diffraction peak splitting [47,48]. Notably, increasing ethanol concentration promoted the formation of highly aggregated and crystalline structures in the KGM gel matrix.

### 2.7. Thermal Gravimetric Analysis (TGA) Analysis

**Figure 7:**
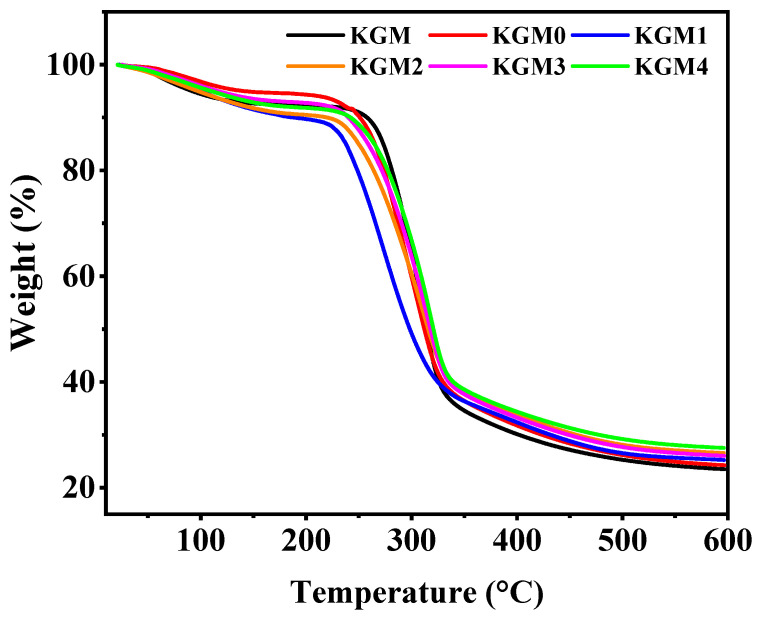
The TGA curve of KGM gels with different ethanol concentrations (0–20% *v*/*v*). KGM0, KGM1, KGM2, KGM3, and KGM4 correspond to gels prepared with final ethanol concentrations of 0, 5, 10, 15, and 20% (*v*/*v*), respectively. KGM correspond to polymer powder alone.

The TGA curves (Figure 7) reveal the effect of ethanol on the thermal degradation behavior and thermal stability of the KGM gels. TGA provided evidence for the thermostability of the ethanol-induced structural rearrangements in KGM gels. The samples underwent three-stage thermal degradation, including a water evaporation stage at 20–250 °C, a main chain degradation stage at 250–350 °C, and a high-temperature carbonation stage at >400 °C [48,49]. This degradation pattern of polysaccharide gels indicates the loss of physically bound water, breakdown of the polysaccharide backbone, and formation of residues. The weight-loss rate of the samples in the low-temperature region (20–250 °C) decreased with increasing ethanol concentration. This weight-loss corresponds to the reduction in the number of free and bound water molecules within the gel matrix. This indicates that the addition of ethanol enhances the intermolecular hydrogen bonding and hydrophobic interactions, which reduces the free water content and allows the water to exist in a more bound state, suppressing mass loss at low temperatures. In the main degradation interval (250–350 °C), the onset temperature of thermal decomposition increased with increasing ethanol concentration. This delayed decomposition indicates the improved resistance of the ethanol-assisted KGM gel to thermal decomposition. Ethanol improved activation energy of thermal decomposition of the gels by promoting ordered chain packing and network densification [50], which made the system more thermally stable. In addition, at the high-temperature carbonization stage (>400 °C), the residual carbon rate was slightly elevated with an increase in the ethanol concentration. This may be related to the enhancement of the crosslinking density, which made the high-temperature residue after pyrolysis more stable. These results suggest that the addition of ethanol improves the thermal stability of the KGM gels, enabling them to exhibit greater resistance to thermal degradation in high-temperature environments. Ethanol-equilibrated KGM gels exhibited higher onset thermal decomposition temperatures than the aqueous control, indicating enhanced thermal stability due to increased chain aggregation and reduced hydration, which require greater energy for thermal decomposition [15]. Although the addition of 20% *v*/*v* of ethanol partially disrupted the gel network, the reduction in free water during heating enhanced hydrophobic interactions, leading to tighter molecular binding and good thermal stability of the sample.

### 2.8. Scanning Electron Microscope (SEM) of a KGM Gels

**Figure 8:**
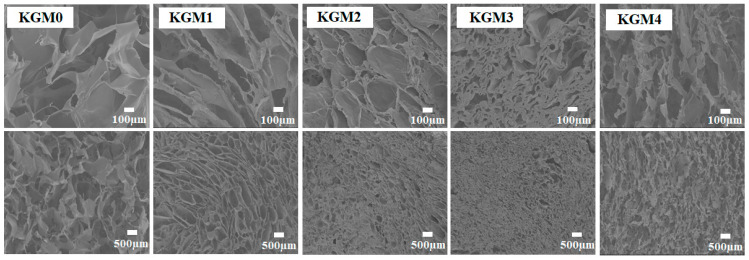
Scanning electronic microscopy images of KGM gels with different ethanol concentrations (0–20% *v*/*v*) at scales of 100 µm and 500 µm. KGM0, KGM1, KGM2, KGM3, and KGM4 correspond to gels prepared with final ethanol concentrations of 0, 5, 10, 15, and 20% (*v*/*v*), respectively.

In view of the key role of polymer microforms in structural analysis, we further visualized the microstructure of KGM gels. As shown in Figure 8, the KGM gel exhibited a typical honeycomb 3D network structure. This honeycomb structure is characteristic of polysaccharide and protein-based gel systems and is crucial for WHC and mechanical strength. A similar honeycomb structure has been observed in various gels, such as gelatin methacryloyl hydrogels, pea-protein/hydroxypropyl starch hydrogels, and agar gels [51,52,53]. In the absence of ethanol, KGM0 displayed a loose and porous network structure with large internal cavities. With increasing ethanol concentration, the pore sizes of KGM1, KGM2 and KGM3 gels gradually decreased, and the structures became increasingly continuous and dense. This increased reduction in pore size indicates enhanced physical crosslinking and improved structural uniformity. This is attributed to the fact that as the concentration of ethanol increases, the hydrophobic interactions become stronger and the free rotational space of the molecular chains becomes smaller [11,54]. Chain segments are required to traverse the spatial domains occupied by neighboring chains during motion, resulting in extensive interchain entanglement that facilitates the formation of a continuous gel network. However, with further increases in ethanol concentration, the KGM4 gel network showed partial structural disruption, as evidenced by the increase in pore size and loosening of the network structure. This phenomenon may be attributed to the high concentration of ethanol, which disrupts the dynamic equilibrium between hydrophobic interactions and hydrogen bonding. Excessive hydrophobic interactions led to excessive aggregation and entanglement of the molecular chains, which destroyed the structural integrity of the gel network. This result is consistent with the results of the variation in ethanol concentration [41,55]. Overall, SEM analysis provided visual evidence for the optimal ethanol concentration for KGM gel formation, further confirming the optimal ethanol concentration identified through WHC, rheological structure, and thermal analysis.

## 3. Conclusions

In this study, high-performance KGM gels were successfully prepared by regulating the ethanol concentration under low-alkali conditions. The results demonstrated that under low-alkali conditions, the addition of a moderate amount of ethanol (0–20% *v*/*v*) promoted the ordered stacking of KGM molecular chains, induced the rearrangement of hydrogen bonds, and enhanced hydrophobic interactions. The mechanical strength, thermal stability, and density of the gel network structure were significantly improved. However, the addition of excessive ethanol led to excessively strong intermolecular interactions among the KGM chains, resulting in localized aggregation and disruption of the gel network structure. Comprehensive analysis revealed that 15% *v*/*v* of ethanol exhibited the strongest synergistic effect among the tested conditions between hydrogen bonding and hydrophobic interactions in KGM gels, resulting in the best mechanical properties. This study shows that controlled ethanol incorporation regulates the hydration, intermolecular interactions, and network structure of KGM gels, establishing structure–property relationships that provide a theoretical basis for designing ethanol-containing semi-solid food products with tailored properties.

## 4. Materials and Methods

### 4.1. Materials

KGM was purchased from Hubei Yizhi Konjac Industry Co., Ltd. (Yichang, China) (purity ≥ 92%, molecular weight 1.4 MDa, degree of acetylation 5–10%). The ethanol used in this study was analytical grade ethanol (≥99.7% purity) supplied by Shanghai McLean Biochemical Technology Co., Ltd. (Shanghai, China). Sodium carbonate (Na_2_CO_3_, ≥99.8%) was provided by Sinopharm Chemical Reagent Co., Ltd. (Shanghai, China). All chemicals used in this study were of analytical grade.

### 4.2. Preparation of KGM Gels

The KGM powder was slowly dispersed in ultrapure water to form a 1% (*w*/*v*) KGM sol. The ultrapure water was prepared through a Milli-Q water purification system (Millipore, Burlington, MA, USA). The KGM solution was divided into 20 mL aliquots, and 1 mL of Na_2_CO_3_ solution with a concentration of 0.8% (*w*/*v*) was mixed and a standard magnetic stirrer was used to ensure homogeneous dissolution of KGM for 3 min. The pH of the solution was 9.6. Ethanol was added to the homogeneous KGM-Na_2_CO_3_ solution to reach the final ethanol concentrations of 0, 5, 10, 15 and 20% (*v*/*v*), respectively, followed by mixing using a standard magnetic stirrer for 5 min to obtain a homogeneous mixture. Finally, the solution was stored in a water bath at 70 °C for 30 min to form a gel and then stored overnight at 4 °C. Each gel formulation was independently prepared in triplicate (three independent batches). The prepared KGM gels were sequentially named KGM0, KGM1, KGM2, KGM3, KGM4.

### 4.3. Determination of Swelling Ratio

A modification of the classical gravimetric method of Sason and Nussinovitc was used to measure the swelling ratio of the gel [10]. Freshly prepared gels were first freeze-dried until a constant weight was achieved to remove all free and bound water. The mass of the dried gel was recorded as m_0_. The samples (m_0_) were placed in deionized water for swelling at 25 °C to initiate swelling. The gels were removed at regular intervals (0–50 h, at 10 h intervals) and weighed again (m_t_) by wiping off excess water with filter paper, and swelling was terminated until a constant weight was reached. The gel swelling ratio was calculated using Equation (1).

(1) $$Swelling\;ratio\;(\%)=\frac{m_{t}-m_{0}}{m_{0}}\times 100$$

### 4.4. Measurement of WHC

A total of 3 g of freshly prepared gels (containing their initial water) were loaded into a pre-weighed centrifuge tube (m_0_), and the tube was weighed again (m_1_). The gel was placed in a centrifuge (TG16-WS, Xiang Yi Instrument Co., Ltd., Changsha, China) and centrifuged at high speed (8000 rpm, 20 min, at 25 °C) and weighed after removing the water (m_2_). The released liquid was considered to contain predominantly water. WHC measurements primarily reflect the integrity and water-retention ability of the gel network rather than the absolute ethanol content retained in the gel.

Equation (2) was used to calculate gel water-holding capacity.

(2) $$WHC\;(\%)=\frac{m_{2}-m_{1}}{m_{1}-m_{0}}\times 100$$

### 4.5. Texture Profile Analysis (TPA)

The textural properties of the gels were determined using the TPA mode of a texture analyzer (TMS-Pro 300, FTC, Sterling, VA, USA) at 25 °C. Cylindrical gel specimens (30 mm diameter × 25 mm height) were prepared and placed centrally on the analyzer platform. A cylindrical compression probe with a diameter of 40 mm was used for the measurements. The instrument was set up with the following parameters: range, 250 N; compression ratio, 50% of the original sample height; trigger force, 0.1 N; and test speed, 30 mm/min, and intermission time of 5 s between the two compression cycles.

### 4.6. Rheological Properties Measurements

The rheological characteristics of KGM gels (KGM0, KGM1, KGM2, KGM3 and KGM4) prepared according to Section 4.2 were examined using a DHR-2 rheometer (TA Instruments, New Castle, DE, USA) fitted with a 40 mm plate and a 5 mm measuring gap at 20 °C. The apparent viscosity was evaluated at shear rates ranging from 0.1 to 100 s^−1^. Frequency sweeps were performed at a constant strain of 1% over an angular frequency range of 0.1–100 rad/s. The dynamic temperature sweep was assessed from 25 to 70 °C with a heating rate of 5 °C/min and a frequency of 1 Hz. During the measurement, one or two drops of silicone oil were placed on the edge of the plate to prevent water loss due to gel evaporation [56].

### 4.7. Determination of FTIR

The gel samples were frozen at −80 °C for (12–24 h) and subsequently freeze-dried using a laboratory freeze dryer under vacuum conditions for (48 h). Subsequently, the samples were milled into powder. The FTIR spectra of the powdered samples were obtained using a Fourier transform infrared spectrometer (PerkinElmer, Norwalk, CT, USA) with the pressure-slice (KBr pellet) method to determine their structural characteristics. The scanning was performed in the wavenumber range of 4000 to 500 cm^−1^ with a resolution of 4 cm^−1^.

### 4.8. Measurement of XRD

XRD patterns of the freeze-dried gels were obtained at room temperature (25 °C) using an X-ray diffractometer (Smartlab9, Tokyo, Japan) Cu Kα radiation source (λ = 1.5406 Å). The scan data were recorded in an angular (2θ) range of 5° to 50° at a scan rate of 10°/min.

### 4.9. Determination of TGA

Thermogravimetric analysis (TGA, SDT Q600, TA Instruments, USA) was employed to assess the thermal stability of the gel samples. Approximately 7–13 mg of each specimen was placed in a platinum sample pan and subjected to heating from 20 °C to 550 °C at a constant rate of 10 °C per minute under nitrogen flow, and the corresponding mass loss was continuously monitored as the temperature increased.

### 4.10. Microstructural Observations

The microstructure of the freeze-dried and powdered gels, as described in Section 4.7, was examined by scanning electron microscopy (SEM; Hitachi S-4800, Tokyo, Japan). Freeze-dried samples were mounted on aluminum stubs with a conductive adhesive and coated with a thin layer of platinum to enhance conductivity. Microphotographs were captured at magnifications corresponding to 100 µm and 500 µm scales, under an accelerating voltage of 3 kV.

### 4.11. Statistical Analysis

All results are presented as mean ± standard deviation (SD) based on triplicate measurements and plotted using Origin 2021. IBM SPSS Statistics 22 (IBM Corp., Armonk, NY, USA) was selected for statistical analysis of the data, and Duncan’s Multiple Range Test was applied to determine significance at *p* < 0.05.

## Data Availability

The original contributions presented in this study are included in the article. Further inquiries can be directed to the corresponding authors.
